# Method for Extracting Impact Signals in Falling Weight Deflectometer Calibration Based on Frequency Filtering and Gradient Detection

**DOI:** 10.3390/s25113317

**Published:** 2025-05-24

**Authors:** Jiacheng Cai, Yingchao Luo, Bing Zhang, Lei Chen, Lu Liu

**Affiliations:** 1 Research Institute of Highway, Ministry of Transport, Beijing 100088, China; jc.cai@rioh.cn (J.C.); b.zhang@rioh.cn (B.Z.); 2 China-Road Transportation Verification & Inspection Hi-Tech Co., Ltd., Beijing 100088, China; chenlei940416@163.com

**Keywords:** FWD, impact point identification, frequency domain filtering, gradient detection, signal reconstruction

## Abstract

FWD is an important non-destructive testing instrument in the field of highways. It evaluates the pavement bearing capacity by continuously hammering the ground. However, due to noise interference, the current identification and extraction of the impact signals generated by the hammering are not accurate enough, which affects the calibration accuracy of the FWD results. To address this issue, this work proposes a novel method for impact point identification. The method integrates frequency domain filtering with gradient detection. Firstly, by analyzing the frequency domain characteristics of FWD impact signals using fast Fourier transform (FFT) and short-time Fourier transform (STFT), the primary response frequency band of the impact was identified. Subsequently, the impact signal segment was reconstructed using inverse fast Fourier transform (IFFT) to effectively suppress noise interference. Furthermore, gradient detection was employed to precisely determine the initiation moment of the impact. To validate the proposed method, a simulated acceleration signal incorporating interference noise was constructed. Comparative experiments were also conducted between traditional identification methods and the proposed method under high-noise conditions. The results demonstrate that the proposed method can accurately identify the impact point even under strong noise, thereby providing reliable data support for FWD measurements. This method exhibits strong environmental adaptability and can be extended to other engineering tests involving impact events and impact point identification.

## 1. Introduction

The FWD is a widely used non-destructive testing device in road engineering and is primarily employed to assess the structural bearing capacity of pavements. Its working principle involves simulating vehicle loads by applying impact forces to the pavement, which causes pavement deflection and indirectly assesses the pavement’s quality and structural performance [1,2]. Specifically, the FWD device applies a rated impact force to the pavement by lifting and releasing a weight of a certain mass, and sensors record the pavement’s response. These signals support the inverse analysis of parameters such as the elastic modulus and stiffness of the pavement materials [3,4,5], thus becoming core data in road quality assessments [6,7,8,9,10].

The accuracy of FWD measurement results has always been a key concern in the field of highway engineering. Research has shown that the repeatability of measurements from a single FWD is generally good, but that there are significant differences in the measurement results between multiple FWDs [11,12]. Currently, the FWD has been employed in comparative tests with laser high-speed deflectometers [13], highlighting the stringent requirements for the accuracy of FWD measurements. Therefore, in-depth research into FWD calibration technology is necessary to ensure the reliability and consistency of measurement results. Currently, due to differing technical focuses, various calibration methods are being explored. High-frequency Doppler laser interferometers and a rated mass hammer can trace the value of the FWD’s impact force sensors [14,15,16]. Although this method has high precision, it has limitations in terms of response. These methods require the disassembly of impact force sensors from the original equipment, which alters the equipment’s state before and after the traceability test. As a result, the research of in-situ calibration technology, which does not require disassembling the sensors, is urgently needed [17,18].

To ensure the accuracy of FWD measurement results, accelerometers are typically used as references, with the accelerometer and the seismic detector being co-axially arranged for calibration [19]. The reference device collects impact signals and identifies the impact point based on the amplitude and standard deviation of the acceleration [20]. However, during actual operations, the FWD device is prone to mechanical vibration interference during calibration, leading to errors in impact point identification. On the one hand, vibration interference may be misidentified as an impact signal, and on the other hand, the impact point identified by the reference device may be significantly different in time from the true impact point. Identifying the impact point in impact signals is crucial for the accuracy of acceleration integration [21]. If the impact point is misjudged, it directly affects the precision of subsequent integration calculations, which in turn impacts the evaluation of the pavement’s structural bearing capacity. Differences in algorithm definitions can lead to significant discrepancies in measurement results [22]. Therefore, improving the accuracy of impact point identification in impact signals is key to solving this issue.

To address this problem, this work proposes a novel impact point identification method that integrates frequency domain filtering and gradient detection. First, through FFT and STFT, the amplitude–frequency characteristics of hundreds of FWD impact signals were statistically analyzed, and the primary frequency band of the impact signal was identified. After determining the target frequency band, the signal was reconstructed using IFFT to eliminate noise interference. When non-stationary signals are affected by noise, the amplitude of the signal within the target frequency band becomes difficult to estimate [23,24,25,26,27,28,29,30]. In this work, gradient detection was employed to locate the precise start time of the impact by detecting abrupt changes in the signal. This method effectively suppresses noise interference and accurately identifies the impact point in FWD signals, greatly improving the accuracy and reliability of the device calibration.

The contribution of this work lies in the proposal of a new signal processing method that combines frequency domain filtering and gradient detection, providing an effective solution for reducing impact point errors in FWD impact signal identification during calibration tests. Experimental results show that the proposed method can still accurately identify the impact point under high noise conditions, providing more reliable data support for FWD calibration and demonstrating strong environmental adaptability.

## 2. FWD Calibration Methods and Signal Features

### 2.1. Experimental Principle

**Figure 1:**
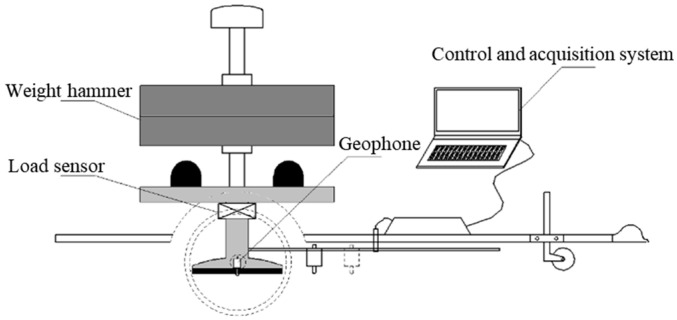
Schematic diagram of FWD structure.

The FWD primarily consists of a weight hammer, load sensor, geophone, and control/acquisition system, as illustrated in Figure 1. Once the weight hammer is lifted and released, the load sensor ensures that the hammer generates the rated impact force necessary for measuring the pavement structure by providing feedback on the force. After the geophone collects the velocity signals, the control and acquisition system performs integration operations to obtain the displacement sequence, which is then used to determine the pavement deflection.

**Figure 2:**
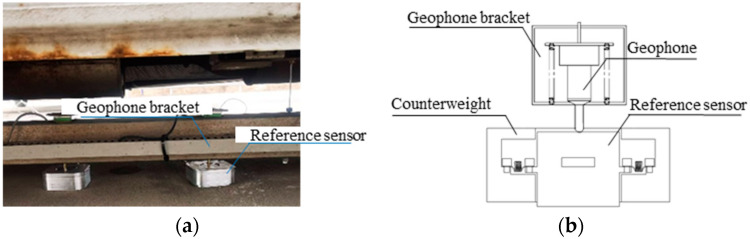
Sensor layout in calibration tests. (**a**) Physical layout of sensors and (**b**) schematic diagram of mechanical structure.

The calibration of the FWD geophone (Dynatest A/S, Ballerup, Denmark) typically uses an accelerometer as the reference sensor. The accelerometer is designed as a freely movable unit module, and the arrangement of the accelerometer and geophone as well as the mechanical structure are shown in Figure 2a,b. The accelerometer is positioned in the middle as a movable unit, connected to the surrounding counterweight via springs, and remains in close contact with the ground. The probe of the geophone is pressed against the accelerometer, simulating the measurement conditions of the geophone on the ground to the greatest extent. This sensor layout avoids the need to disassemble the geophone during calibration tests, enabling in-situ calibration of the FWD. During the calibration process, the accelerometer collects the impact signals, and, through processing algorithms, the impact point is identified. Afterward, the displacement sequence is obtained through two integrations, with the maximum displacement serving as the deflection reference value.

### 2.2. Traditional Identification Methods

**Figure 3:**
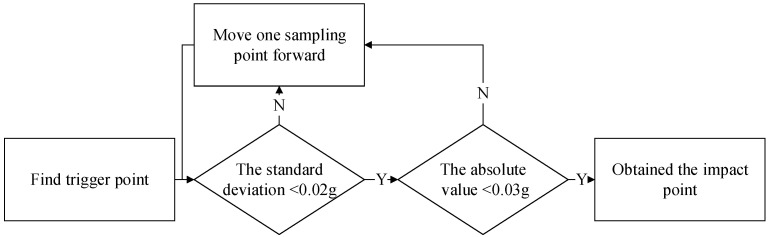
Traditional impact point identification algorithms.

In the calibration test of the FWD, the accelerometer continuously collects acceleration signals, and a signal processing algorithm is used to identify the occurrence of the hammer impact event. In traditional algorithms for impact point identification, the process begins by setting a threshold of 0.2 g to initially filter out potential impact triggers. After detecting this trigger point, the algorithm then checks forward in the signal. It is typically assumed that, if the standard deviation of the next 25 consecutive samples does not exceed 0.02 g and the absolute value of the acceleration remains below 0.03 g, then the current sample point is identified as the impact point [19]. The algorithm flow is shown in Figure 3.

**Figure 4:**
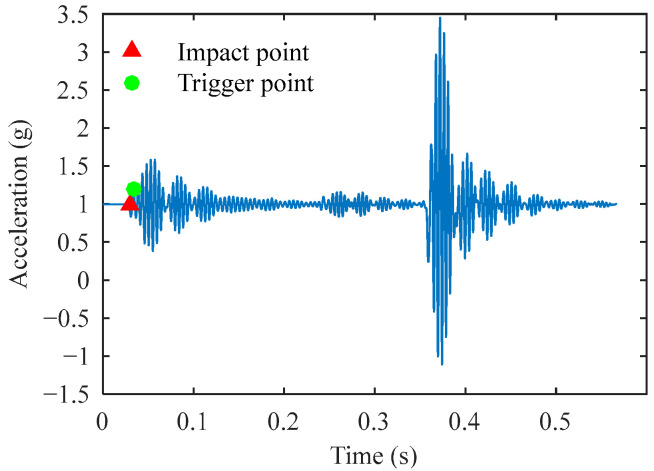
Misidentification of trigger and impact points caused by interference signals in traditional algorithms.

Although this method is simple, its effectiveness significantly decreases in experimental environments with high levels of interference or noise. The most obvious issue with traditional algorithms is the use of a 0.2 g acceleration signal fluctuation as the criterion for triggering the impact event. When the interference signal exceeds the threshold, it triggers the subsequent algorithm steps. As shown in Figure 4, when the interference signal amplitude exceeds the threshold, the traditional algorithm identifies both the trigger point and the impact point. The abrupt change in the signal here is caused by mechanical vibrations during the FWD hammer lifting, while the actual impact occurs after 0.3 s. In this case, it may not be possible to accurately calculate the reference deflection. Furthermore, in calibration tests, it is challenging for the operators to ensure that the reference deflection value output by the signal processing program is derived from a valid impact signal.

### 2.3. Frequency Spectrum Characteristics of the Impact Signal

**Figure 5:**
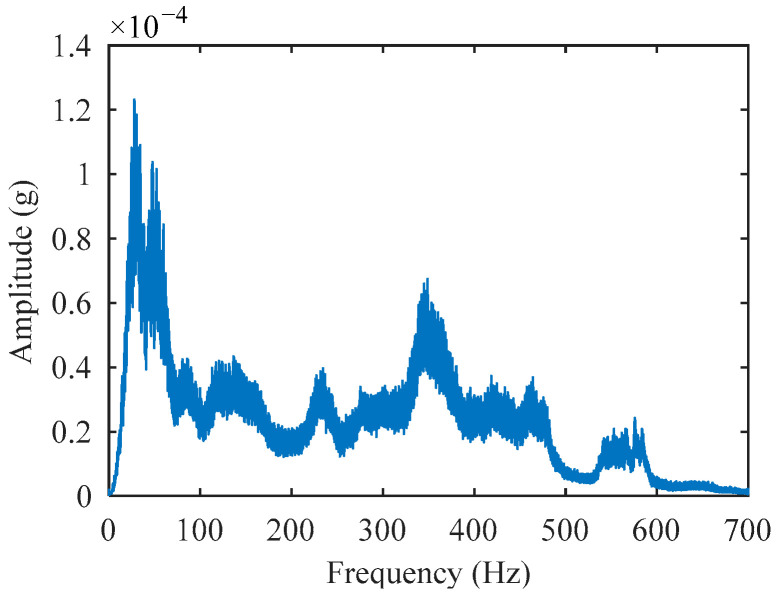
Average amplitude–frequency spectrum.

To address the aforementioned misidentification issue, noise reduction processing is necessary for the acceleration impact signals. To obtain statistically significant signal amplitude–frequency characteristics, this work performed FFT on nearly 500 impact signals and calculated the average amplitude at each frequency point. The data were collected from falling weight calibration tests. These tests were conducted by the authors and research team members. The experimental sites included five major cities: Beijing, Guangzhou, Chongqing, Xining, and Urumqi. To ensure data validity, a strict screening criterion was applied. Only impact sequences with deflection values showing ≤2% relative standard deviation were selected. To accurately identify impact events, it is essential to select a frequency band that is as narrow as possible while simultaneously ensuring this band exhibits the most significant response characteristics to the impact. As shown in Figure 5, the maximum amplitude of the signal occurs within the frequency band of 10 Hz to 40 Hz.

**Figure 6:**
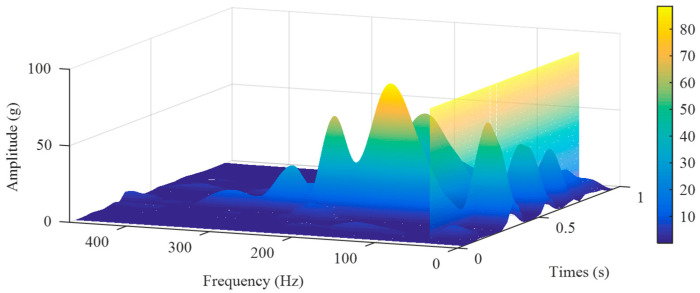
STFT analysis of a typical signal.

After confirming the significant contribution of the 10~40 Hz frequency band to the impact signal’s amplitude, we need to investigate whether the frequency components within this band exhibit a sufficiently significant response to the sudden change of the impact. Therefore, in this work, the typical experimental data were analyzed using STFT, and the results are shown in Figure 6. The frequency corresponding to the vertical plane is 40 Hz. It can be observed that the peak of the frequency components in the 10~40 Hz range aligns perfectly with the actual impact events on the time scale, confirming that the signal frequencies within this frequency band have a significant response to the FWD falling hammer impact.

## 3. Frequency Domain Filtering and Impact Point Identification Method

### 3.1. Frequency Domain Filtering

To accurately identify the trigger and impact points in the presence of interference signals, this work proposes an impact point identification algorithm that combines frequency domain filtering and gradient detection, serving as a replacement for the traditional method that solely relies on acceleration thresholding.

For a simulated signal x(t) that changes with time t, its discrete signal expression obtained at the sampling frequency $f_{s}$ is given by

(1) $$x[n]=x(t_{n}),t_{n}=n\cdot \Delta t,n=0,1,2,\ldots ,N-1$$

In Equation (1), n is the index of the discrete signal, $t_{n}$ represents the discrete time sequence, $\Delta t=\frac{1}{f_{s}}$ is the sampling interval, and *N* is the total number of sampling points.

In most scenarios of amplitude–frequency analysis, we typically calculate the complete spectrum of the signal, determining the amplitude and phase information at all frequency points. However, the goal of this method is to obtain only partial amplitude–frequency information. Therefore, to improve computational efficiency, we perform the amplitude–frequency conversion only on the target frequency band. The frequency domain representation of the original signal x[n] is given by the following equation:

(2) $$X[k]=\sum_{n=0}^{N-1}\;x[n]\cdot e^{-j2\pi \frac{k}{N}n},k=0,1,2,\ldots ,N-1$$

In Equation (2), k is the index of the frequency point and X[k] is the amplitude and phase of the signal x[n] at the frequency $f_{k}$, where $f_{k}=\frac{k}{N}f_{s}$ is the frequency sequence.

In order to perform band-limiting, a target frequency range $\left[f_{\mathrm{low}},f_{\mathrm{high}}\right]$ needs to be specified. Based on the conclusions from the previous section, $f_{\mathrm{low}}$ and $f_{\mathrm{high}}$ are set to 10 Hz and 40 Hz, respectively. The frequency components within this range are fully preserved, while the frequency components outside this range are set to zero. The indices of the lower and upper frequency limits of the band are calculated based on Equations (3) and (4).

(3) $$k_{\mathrm{low}}=\left\lfloor \frac{f_{\mathrm{low}}}{f_{s}/N}\right\rfloor$$

In Equation (3), $k_{\mathrm{low}}$ is the index corresponding to the lower frequency limit  $f_{\mathrm{low}}$.

(4) $$k_{\mathrm{high}}=\left\lfloor \frac{f_{\mathrm{high}}}{f_{s}/N}\right\rfloor$$

In Equation (4), $k_{\mathrm{high}}$ is the index corresponding to the upper frequency limit  $f_{\mathrm{high}}$.

Therefore, for each frequency point k within the target frequency band, its Fourier transform is calculated according to Equation (5).

(5) $$X_{t}\left[k\right]=\sum_{n=0}^{N-1}x\left[n\right]\cdot e^{-j2\pi \frac{k}{N}n},k=k_{\mathrm{low}},k_{\mathrm{low}}+1,\ldots ,k_{\mathrm{high}}$$

In Equation (5), $X_{t}\left[k\right]$ is the amplitude and phase of the target frequency band.

After setting the other frequency components to zero, the frequency domain representation of the signal’s positive frequency part is given by Equation (6).

(6) $$X_{p}[k]=\left\{\begin{array}{ll} X_{t}[k], & k_{\mathrm{low}}\leq k\leq k_{\mathrm{high}}\\ 0, & 0\leq k<k_{\mathrm{low}}{,k}_{\mathrm{high}}<k\leq \frac{N}{2} \end{array}\right.$$

In Equation (6), $X_{p}[k]$ is the amplitude and phase of the positive frequency part of the signal to be reconstructed.

To obtain a real-valued reconstructed signal, the positive and negative frequency components in the frequency domain need to satisfy conjugate symmetry. Therefore, the frequency domain representation of the signal to be reconstructed is given by Equation (7).

(7) $$X[k]=\left\{\begin{array}{ll} X_{p}[k], & 0\leq k\leq \frac{N}{2}\\ X_{p}^{*}[N-k], & \frac{N}{2}<k\leq N-1 \end{array}\right.$$

Finally, performing the IFFT on X[k] completes the signal reconstruction, and its expression is given by Equation (8):

(8) $$\hat{x}[n]=\frac{1}{N}\sum_{k=0}^{N-1}X_{p}\left[k\right]\cdot e^{j2\pi \frac{k}{N}n}$$

In Equation (8), $\hat{x}[n]$ is the reconstructed time–domain signal.

### 3.2. Gradient Detection

After the signal is reconstructed, the amplitude variation in response to the impact event becomes very significant, creating the conditions for accurate impact point identification. As the target frequency band does not contain all the impact information, it is difficult to identify the impact point using only the acceleration threshold method. Therefore, a gradient detection method is used to identify the impact point. To eliminate amplitude differences under different experimental conditions, the reconstructed signal is normalized. The normalization process scales the signal’s amplitude to a uniform range, making it easier for subsequent impact point identification and analysis. The normalized reconstructed signal is calculated according to Equation (9).

(9) $${\hat{x}}_{\mathrm{nor}}\left[n\right]=\frac{\hat{x}\left[n\right]-\mu }{\sigma }$$

In Equation (9), ${\hat{x}}_{\mathrm{nor}}\left[n\right]$ is the normalized signal, $\mu =\frac{1}{N}\sum_{n=1}^{N}\hat{x}\left[n\right]$ is the mean of the signal, and $\sigma =\sqrt{\frac{1}{N-1}\sum_{n=1}^{N}{\left(\hat{x}\left[n\right]-\mu \right)}^{2}}$ is the standard deviation of the signal.

In Equation (10), the forward difference sequence of the normalized signal is calculated. Based on engineering experience, a gradient threshold of 0.02 is set to determine the trigger point. The recognition of the trigger points in the above process is performed on the reconstructed signal, which involves frequency domain truncation during the signal reconstruction process. This theoretically leads to a certain degree of phase shift. In the previous section, the frequency domain selection was made between 10 Hz and 40 Hz, which, due to its low frequency and relatively large bandwidth, results in a very minimal phase shift effect. To further reduce the phase impact, after the trigger point index is obtained from the normalized signal, a standard deviation and threshold check is performed on the original signal from the same index of the forward sampling points to determine the final impact point.

(10) $$∆{\hat{x}}_{\mathrm{nor}}\left[n\right]={\hat{x}}_{\mathrm{nor}}\left[n+1\right]-{\hat{x}}_{\mathrm{nor}}\left[n\right]$$

**Figure 7:**
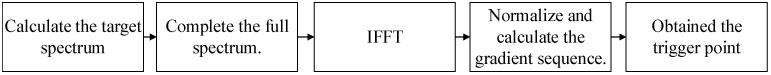
Frequency domain filtering and gradient detection algorithm flow.

Therefore, the difference between this method and the traditional impact point identification method lies in the frequency domain filtering and the gradient detection algorithm for trigger points. The algorithm flow for this part is shown in Figure 7.

## 4. Simulation Signal and Identification Results

In the FWD impact on the pavement, the pavement will oscillate multiple times in both the forward and reverse directions, with the amplitude gradually decaying until it disappears. Under the assumption of neglecting the pavement’s plastic deformation, the pavement can be modeled as an underdamped second-order system [31,32], and the impact input of the FWD can be simulated using a pulse input, from which the time–domain response can be obtained [33,34]. The simulated impact signal is obtained by scaling the time–domain response. The impact simulation signal is given by Equation (11).

(11) $$y\left(t\right)=k\frac{\omega_{n}}{\sqrt{1-\zeta^{2}}}e^{-\zeta \omega_{n}t}\sin\left(\omega_{d}t\right)$$

In Equation (11), $\omega_{d}=\omega_{n}\sqrt{1-\zeta^{2}}$ is the damped natural frequency and k is the scaling coefficient. To simulate the actual signal, k is set to 0.021, $\omega_{n}$ is set to 50π, and ζ is set to 0.2.

The simulation signal needs to incorporate two types of interference noise: one simulates the high-frequency mechanical vibrations generated during the lifting of the FWD hammer, with a larger amplitude and occurring before the impact of the hammer in terms of timing; the other simulates the constant high-frequency background noise. The two types of interference noise are given by Equations (12) and (13).

(12) $$n_{1}(t)=A_{1}sin\;(2\pi f_{1}t)$$

(13) $$n_{2}(t)=A_{2}sin\;(2\pi f_{2}t)$$

In Equations (12) and (13), $n_{1}(t)$ and $n_{2}(t)$ represent the first and second types of interference noise, respectively; $A_{1}$ and $A_{2}$ are the signal amplitudes, with values of 1.5 g and 0.5 g, respectively; and $f_{1}$ and $f_{2}$ are the signal frequencies, with values of 100 Hz and 200 Hz, respectively.

Considering that the acceleration sensor has a reading of 1 g in its natural state, the simulated signal s(t) can be expressed as a piecewise function, as follows:

(14) $$s(t)=\left\{\begin{array}{ll} n_{2}(t)+1, & 0\leq t<0.1\\ n_{1}(t-0.1)+n_{2}(t)+1, & 0.1\leq t<0.12\\ n_{2}(t)+1, & 0.12\leq t<0.32\\ y(t-0.32)+n_{2}(t)+1, & 0.32\leq t<0.62 \end{array}\right.$$

**Figure 8:**
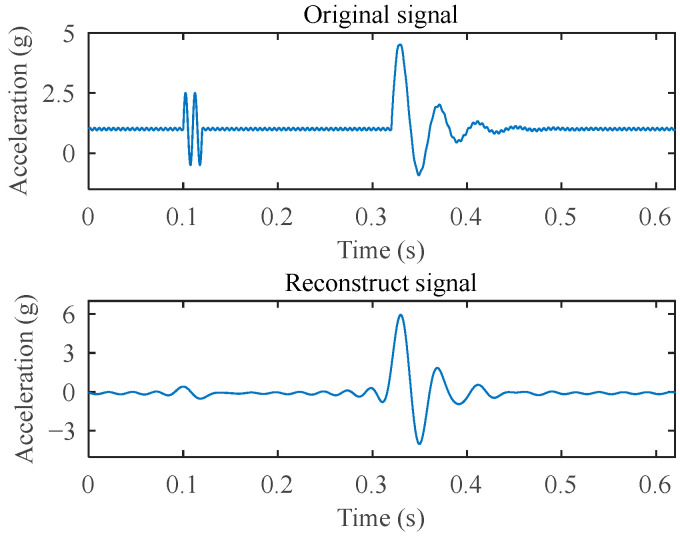
Time–domain comparison of the original signal and the reconstructed signal.

**Figure 9:**
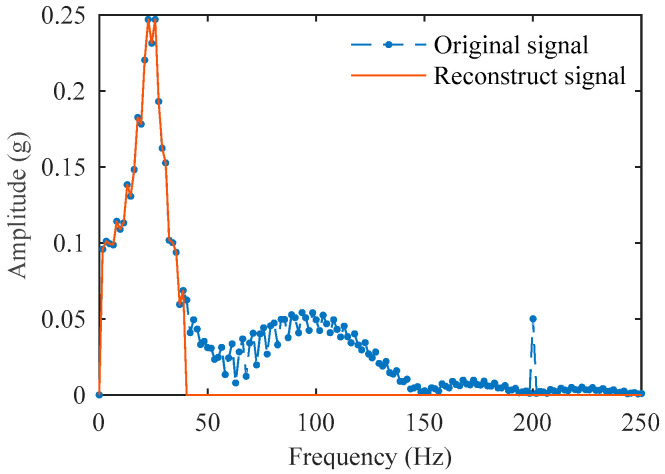
Frequency–domain comparison of the original signal and the reconstructed signal.

This method of synthesizing signals on the timeline intuitively displays the location of the simulated impact point, which is 0.32 s. The sampling frequency of the reference system in the calibration test is set to 15,000 Hz. Under this condition, the comparison of the discrete original simulation signal and its reconstructed signal in both the time domain and frequency domain is shown in Figure 8 and Figure 9. The reconstructed signal significantly suppresses the interference noise, $n_{1}$ and $n_{2}$, outside the target frequency band, while effectively preserving the target impact signal y(t).

**Figure 10:**
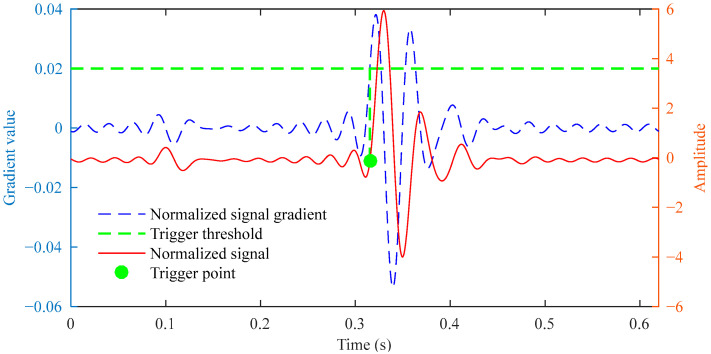
Trigger point identification results of the normalized reconstructed signal.

**Figure 11:**
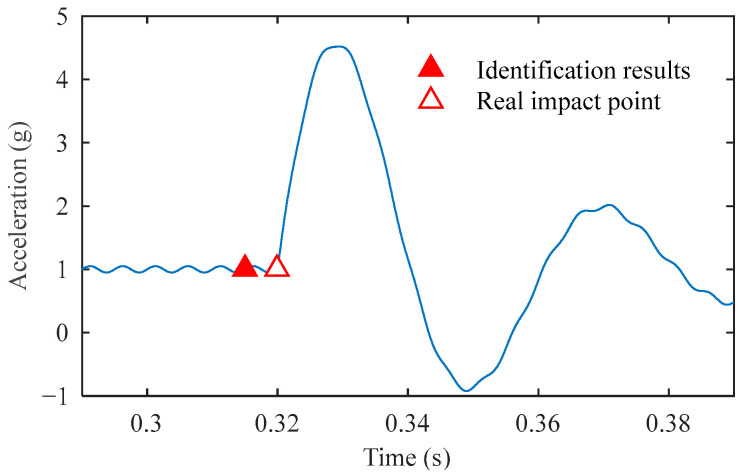
Impact point identification results of the simulated signal.

After normalizing the reconstructed signal and calculating the gradient sequence, the trigger point identification is obtained, as shown in Figure 10. Finally, the impact points identified in the original signal using the standard deviation and acceleration threshold are shown in Figure 11. The algorithm identified the impact point at 0.3151 s, showing merely a 0.0049 s deviation from the preset 0.32 s. This demonstrates that the impact point identification is highly accurate.

The proposed method has quantitatively demonstrated its effectiveness in identifying impact points within simulated signals. However, discrepancies exist between these simulations and actual field measurements under noisy conditions. As shown in Figure 5 and Figure 9, measured signals from real-world environments exhibit dominant amplitudes distributed broadly across the 0~500 Hz range. In contrast, the simulated signals were designed primarily to replicate impact characteristics while incorporating only limited representative noise. Consequently, their significant amplitude range is confined to 0~200 Hz, and their frequency–amplitude distribution patterns differ from those of authentic signals.

## 5. Recognition Results of Impact Signals in Real-World Environments

**Figure 12:**
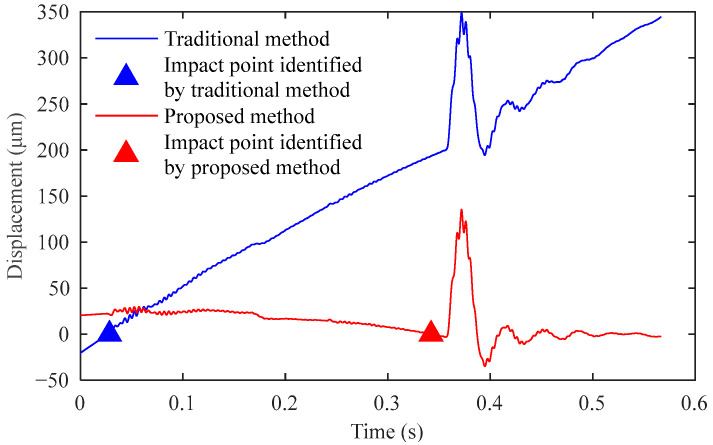
Impact of traditional and proposed methods on displacement sequence and deflection.

In high-noise testing environments, the accuracy of impact point identification significantly affects the final displacement sequence. As demonstrated in Figure 4, noise interference causes traditional methods to identify impact points that deviate substantially from the actual impact locations. As the impact point serves as the starting position for integration calculations, the displacement sequences obtained by traditional methods and our proposed method show marked differences (Figure 12). The traditional approach introduces excessive trend accumulation during integration, resulting in severe displacement sequence distortion. Deflection values are determined by the maximum displacement in the sequence. The traditional method yielded a deflection value of 349.0 μm, while our method produced 135.3 μm. The red signal clearly provides a more accurate representation of pavement dynamic displacement. Under these measurement conditions, the traditional method introduced an additional 213.7 μm of deflection measurement error.

**Figure 13:**
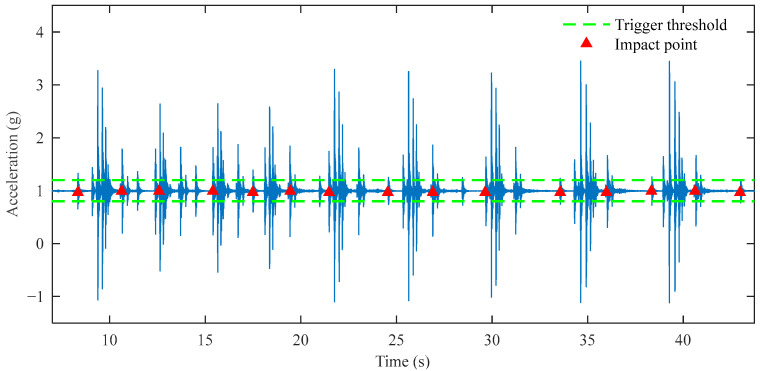
Traditional method impact point identification results in interference noise.

In continuous impact point identification, considering the time duration for the hammer rebound to rest and the mechanical operation time, typically no further impact points are identified within a 2 s window after one impact point has been identified. In actual experiments, when encountering large amounts of interference noise, traditional impact point identification methods become unreliable, and the calculated reference deflection values show poor repeatability. As shown in Figure 13, in this set of experimental data, the mechanical vibration interference has an excessively large amplitude, making it very difficult to identify impact points using traditional methods. The traditional algorithm, based on a 0.2 g threshold, mistakenly identifies mechanical vibrations as impact events, resulting in the identification of 15 impact points. This erroneous identification leads to completely invalid reference deflection values in subsequent integration calculations.

**Figure 14:**
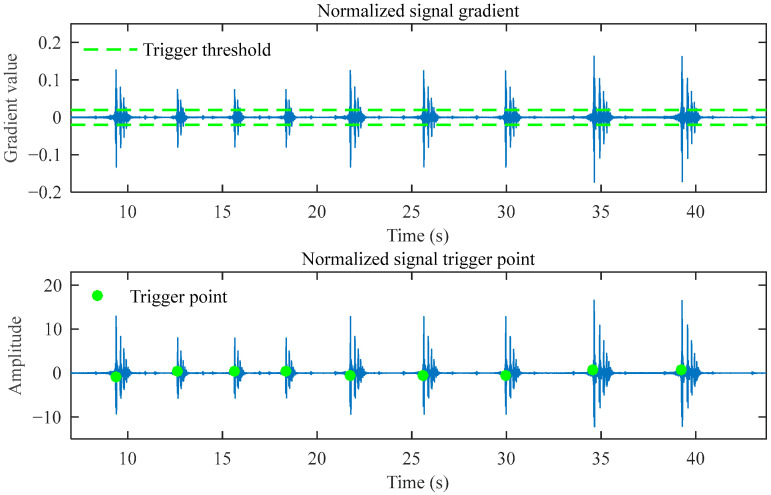
Normalized reconstructed signal trigger point identification results.

**Figure 15:**
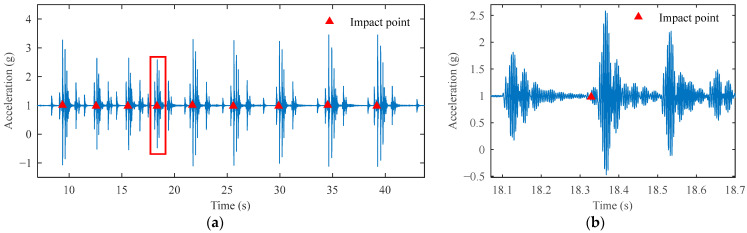
Measured signal impact point identification results. (**a**) All impact point identification results and (**b**) enlarged view of the highlighted segment in (**a**) showing detailed recognition results.

After signal reconstruction, the impact signals become very clear, as shown in Figure 14, with a total of nine impact events corresponding to nine impact trigger points. As shown in Figure 15a, the nine impact points are also accurately identified. In the partially enlarged Figure 15b, multiple peak-decaying impact signals appear after the impact point. These signals correspond to the process where the hammer, after impacting the ground, rebounds and strikes the ground again until it comes to a complete stop. This demonstrates the accuracy and effectiveness of the identification algorithm. Before the impact point, there exists a high-amplitude vibration signal. Based on the operational efficiency of the FWD, this is clearly not a single impact signal. As mentioned earlier, high-frequency vibrations are generated by the equipment during the lifting of the hammer. Our method is immune to such vibration interference, which proves its robustness in real-world testing.

## 6. Discussion

The proposed method demonstrates exceptional performance in simulated signals, achieving an impact point identification error of merely 0.0049 s. However, under real-world testing conditions with substantial noise interference, standalone signal analysis often fails to accurately determine the true impact point. Field environments introduce significantly more complex noise spectra than simulated signals, meaning that the ideal identification accuracy observed in simulations may not always be replicated in practice.

While the target frequency band and gradient threshold were statistically derived from extensive experimental data, variations in pavement structure during calibration tests may necessitate threshold adjustments. To optimize impact point identification, field operators should fine-tune the gradient threshold based on real-time signal characteristics.

For practical deployment across FWDs, calibration systems, and similar impact-based testing equipment, developing a comprehensive validation program becomes essential. This program should integrate sequential pre-impact testing protocols adaptable to varying field conditions. The proposed auxiliary system will provide comparative visualization between gradient sequences and raw signal responses, enabling operators to interactively adjust gradient thresholds and consequently enhance impact point recognition precision.

## 7. Conclusions

This work resolves impact point identification errors in FWD calibration. Through the analysis and statistical processing of nearly 500 impact data using FFT and STFT, the frequency domain characteristics of the FWD impact signal were revealed. It was demonstrated that the signal frequencies in the 10~40 Hz frequency band exhibit significant responses to the FWD hammer impact. Based on this conclusion, a signal reconstruction method based on frequency domain filtering was proposed, which reduced the algorithm’s computational load by only calculating the spectrum of the target frequency band. By normalizing the reconstructed signal, a unified impact recognition threshold was achieved for different amplitude levels. The gradient sequence of the normalized reconstructed signal was calculated, and a gradient threshold was set to identify the trigger points. Finally, combined with the local statistical characteristics of the original signal, the impact points were identified.

This work also proposes a simulation model for FWD calibration experiment impact acceleration signals. The road surface was simplified as an underdamped second-order system, and the hammer impact was simulated by pulse input. The impact signal was simulated by solving the time–domain response. Furthermore, interference noise was added to construct the simulation signal for the calibration experiment. This simulation signal provides a preset impact point for verifying the accuracy of the impact point identification results. After validation, the impact point identified by the proposed method differs from the set value by only 0.0049 s, demonstrating the high precision of the identification results.

In terms of signal reconstruction, validation using simulation signals confirmed that the proposed method effectively retains the target frequency band and suppresses other frequency components as expected. For measured signals containing significant noise, the reconstructed signal clearly showed the actual number of impacts. In terms of impact point identification, the results for both simulated and measured signals accurately displayed the impact points on the time scale. Compared with traditional impact point detection algorithms, this method demonstrated strong robustness against interference noise. In conclusion, the proposed method provides accurate identification results, is highly adaptable to noisy testing environments, and can be extended to other similar engineering tests for precise recognition of impact events and impact points.

## Data Availability

Data will be available on request.

## Abbreviations

The following abbreviations are used in this manuscript:

FWD	Falling weight deflectometer
FFT	Fast Fourier transform
STFT	Short-time Fourier transform
IFFT	Inverse fast Fourier transform
